# Effects of Transcutaneous Auricular Vagus Nerve Stimulation on Peripheral and Central Tumor Necrosis Factor Alpha in Rats with Depression-Chronic Somatic Pain Comorbidity

**DOI:** 10.1155/2020/8885729

**Published:** 2020-10-21

**Authors:** Xiao Guo, Yuanyuan Zhao, Feng Huang, Shaoyuan Li, Man Luo, Yu Wang, Jinling Zhang, Liang Li, Yue Zhang, Yue Jiao, Bin Zhao, Junying Wang, Hong Meng, Zhangjin Zhang, Peijing Rong

**Affiliations:** ^1^Institute of Acupuncture and Moxibustion, China Academy of Chinese Medical Sciences, Beijing 100700, China; ^2^Department of Scientific Research Management, China Academy of Chinese Medical Sciences, Beijing 100700, China; ^3^Department of Psychiatry, First Hospital of Hebei Medical University, Shijiazhuang 050031, China; ^4^Beijing Hospital of Traditional Chinese Medicine, Capital Medical University, Beijing 100010, China; ^5^Key Laboratory of Cosmetic, China National Light Industry, Beijing Technology and Business University, Beijing 100048, China; ^6^The School of Chinese Medicine, LKS Faculty of Medicine, The University of Hong Kong, Hong Kong, China

## Abstract

Depression and pain disorders share a high degree of comorbidity. Inflammatory mechanisms play an important role in the pathogenesis of depression-chronic somatic pain comorbidity. In this study, we investigated the effects of acupuncture on blood and brain regional tumor necrosis factor alpha (TNF-*α*) in rats with depression and chronic somatic pain comorbidity. Forty Sprague-Dawley rats were randomly divided into the following 4 groups with 10 each: control, model, model treated with transcutaneous auricular vagus nerve stimulation (taVNS), and model treated with electroacupuncture (EA). Chronic unpredictable mild stress (CUMS) with chronic constriction injury of the sciatic nerve (CCI) was used to produce depression and chronic somatic pain comorbidity in the latter 3 groups. The rats of the taVNS and EA groups received, respectively, taVNS and EA at ST 36 for 28 days. Pain intensity was measured using a mechanical withdrawal threshold and thermal stimulation latency once biweekly. Depressive behavior was examined using a sucrose preference test at baseline and the end of modeling and intervention. The level of plasma TNF-*α* and the expression of TNF-*α* in the prefrontal cortex (PFC), hippocampus, amygdala, and hypothalamus were measured. While CUMS plus CCI produced remarkable depression-like behavior and pain disorders, EA and taVNS significantly improved depression and reduced pain intensity. CUMS plus CCI also resulted in a significant increase in plasma TNF-*α* level and the expression in all brain regions examined compared to the intact controls. Both EA and taVNS interventions, however, suppressed the elevated level of TNF-*α*. These results suggest that EA and taVNS have antidepressant and analgesic effects. Such effects may be associated with the suppression of TNF-*α*-related neuroinflammation.

## 1. Introduction

Depression is a common mental disorder and often accompanied with unexplained painful physical symptoms [1]. The prevalence of chronic pain is about 51.8% to 59.1% among patients with depression [2]. Pain and depression share a complex reciprocal relationship [3]. (1) More severe depression is accompanied with greater pain. (2) Improvement in pain correlates with improvement in depression [4]. (3) Pain affects the prognosis and treatment of depression, and vice versa [2]. In short, when chronic pain and depression occur concomitantly, the prognosis is worse than in either case, leading to greater functional impairment, longer duration, and less effective medication [5]. The comorbidity increases the socioeconomic cost, and the direct medical costs of comorbid patients are more than twice as high as those with a single disease [3]. Therefore, finding out the underlying mechanisms in order to ensure appropriate treatment and promote the development of new treatments for depression and comorbid pain is urgently needed [6].

Many potential common pathways and neurotransmitters have been proposed to underlie the comorbidity of pain and depression. A growing body of evidences suggested that pain and depression may work in the similar brain regions that manage both mood and noxious sensory pathways of body pains, including the PFC, insular cortex, anterior cingulate, thalamus, hippocampus, and amygdala, which form an anatomical basis for the coexistence of pain and depression [7, 8]. Increasingly, neuroimmune and neuroinflammatory mechanisms are considered to play a key role in the association between depression and pain. An experiment shows that among 37 outpatients with major depression and 48 healthy controls, increased pain sensitivity (by pressure pain thresholds test) in depression may link to increased TNF-*α* concentration [6]. Moreover, in animal models of depression, the increased expression of proinflammatory cytokines in the area of the brain which is responsible for the disposing of emotion and pain is accompanied by inflammation or neuropathic pain [3]. In brief, depression-pain comorbidity is associated with an elevated level of proinflammatory cytokines, including interleukin (IL)-1, IL-6, and TNF-*α* [9]. By affecting chronic pain and depression-related pathophysiological functional areas via the blood-brain barrier, the elevated level of proinflammatory cytokines can result in changes in neurotransmitter metabolism, neuroendocrine function, and neuroplasticity, thus inducing the occurrence of the depression-chronic somatic pain comorbidity [7].

The vagus nerve has been shown to reflexively limit the innate immune response through the binding of its neurotransmitter acetylcholine (Ach) to the *α*7 nicotinic acetylcholine receptor (*α*7nAChR) present on immune cells [10]. Conchae is the only region on our body surface where the vagus nerve innervates. The vagus afferent fibers can project to other brain regions such as the hypothalamus and amygdala via the nucleus tractus solitari (NTS). In consequence, the auricular branch of the vagus nerve is a peripheral pathway to the central nervous system (CNS) [11]. Electric stimuli might follow an inverse path from peripheral nerves toward the brain stem and central structures [12]. Therefore, taVNS can produce a similar effect to the classic vagus nerve stimulation (VNS) on improving the inflammation. Based on the above findings, we postulate an idea that taVNS can improve depression combined with chronic somatic pain by reducing the level of proinflammatory cytokines, such as TNF-*α*. In addition, it was reported that EA at ST 36 can effectively relieve chronic pain [13, 14] and improve depression-like behaviors in rats [15]. Therefore, we select the intervention of EA at ST 36 as the positive control.

## 2. Materials and Methods

### 2.1. Experimental Animals

40 adult male Sprague-Dawley rats (200 ± 20 g) were obtained from China Food and Drug Testing and Research Institute (Beijing, China, Animal License Key No. 2014-0013). They were fed ad libitum and kept at a 23 ± 1°C temperature and 50% ± 10% humidity with an alternating 12 h light/dark cycle. The rats were randomly divided into four groups in conformity to the random digital tables: control group, model group, taVNS group, and EA group, with 10 rats in each group. The rats in the latter 3 groups were single cage rearing. Before modeling, all rats were kept adaptively for seven days. The protocol of the experiment was approved by the Animal Care and Use Committee of the Institute of Acupuncture and Moxibustion, China Academy of Chinese Medical Sciences (D2017-07-31-1).

### 2.2. Instruments, Drugs, and Reagents

Instruments include plantar analgesia meter (IITC Life Science, USA), von Frey filaments (Ugo Basile, Italy), matrix animal anesthesia ventilator system (Midmark, USA), electronic scale (JJ test instrument factory, Changshu, China), HANS instrument (HANS-100A, Nanjing, China), high-speed refrigerated centrifuge (Eppendorf, Germany), mini-protean 3 Dodeca (Bio-Rad, USA), PowerPac HC Power Supply (Bio-Rad, USA), shaker (Kylin-Bell, Haimen, China), homogenizer (IKA, Germany), automatic ice machine (Grant, USA), Multiskan Microplate reader (Thermo, USA), pH meter (Sartorius, Germany), and electrothermal constant temperature incubator (Taisite, Tianjin, China). Glycine, SDS, and Trizma base were purchased from Sigma (Louis, USA). APS, TEMED, Tween-20, Bromphenol Blue, DTT, Acrylamide, and Bis-Acrylamide were obtained from AMRESCO (Washington, USA). Methanol (dried) and NaCl were from Sinopharm (Beijing, China). Goat antirabbit IgG (H+L), HRP, and goat antimouse IgG (H+L), HRP, were obtained from Jackson (West Baltimore Pike, West Grove, PA, USA). Protein lysis buffer was purchased from Ukzybiotech (Beijing, China). BCA Protein Assay Kit was obtained from Beijing Biosynthesis Biotechnology Co., LTD (Beijing, China). The protease inhibitor cocktail was from Roche (Basel, Switzerland). Immobilon ECL was from Millipore (Massachusetts, USA). Nonfat milk was obtained from Dingguo Changsheng Biotechnology (Beijing, China). Isoflurane was purchased from Jiupai (Shijiazhuang, China). The rat TNF-*α* ELISA kit was from Neobioscience (Shenzhen, China). The protein ladder was from Biomed (Beijing, China).

### 2.3. Experimental Procedure

**Figure 1:**
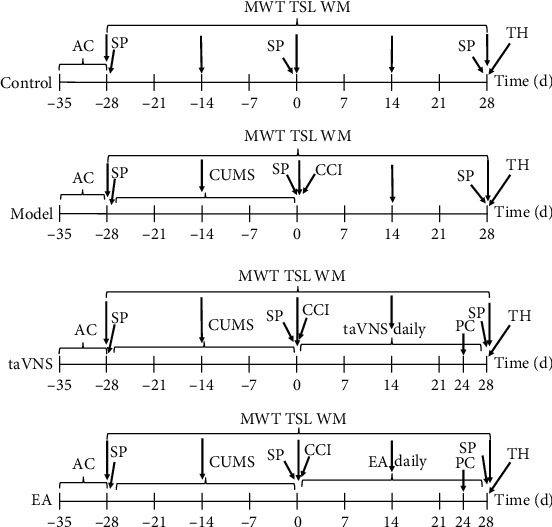
Experimental procedure: AC: acclimatization; SP: sucrose preference; CUMS: chronic unpredictable mild stress; taVNS: transcutaneous vagus nerve stimulation; CCI: chronic constriction injury of the sciatic nerve; MWT: mechanical withdrawal threshold; TSL: thermal stimulation latency; WM: weight measurement; EA: electroaupuncture at ST 36; PC: plasma collection; TH: tissue harvest.

Four groups of rats were given a week of adaptive rearing at -35 day. CUMS was conducted in the latter 3 groups for 28 day. At 0 day, two rats with lower sucrose preference in the control group and two rats with higher sucrose preference in the model, taVNS, and EA groups were, respectively, removed. Then, CCI was performed in the latter 3 groups, with 8 rats in each group. The success of the CUMS combined with CCI model was evaluated by the sucrose preference test, mechanical withdrawal threshold, and thermal stimulation latency. After the modeling is completed, the intervention is carried out. The taVNS and EA groups were intervened for 28 consecutive days, respectively. Plasma and brain tissues in the PFC, hippocampus, amygdala, and hypothalamus were collected in each group at 28 day. The sucrose preference test was performed at -28 day, 0 day, and 28 day. The mechanical withdrawal threshold, thermal stimulation latency, and weight measurement were performed at -28 day, -14 day, 0 day, 14 day, and 28 day. The plasma was taken from the rats in the taVNS and EA groups at 0 min, 15 min, and 30 min to test the immediate effects of the interventions at 24 day. The interventions of taVNS and EA lasted 30 min; therefore, 0 min, 15 min, and 30 min represent before the intervention, during the intervention, and after the intervention, respectively. Unfortunately, two rats from the control and EA groups died accidentally while being taken blood from the tail vein at 24 day (Figure 1).

### 2.4. Chronic Unpredictable Mild Stress Model

The CUMS model is a widely recognized depression modeling method at home and abroad, which can preferably simulate the pathogenesis of human depression. In this experiment, the rats in the latter 3 groups received seven kinds of CUMS, including upside-down day and night (12/12 h), hot plate test (52°C, 5 min), swimming at 8°C–10°C (5 min), in a wet cage (24 h), tail pinch (3 min), food deprivation (24 h), and water deprivation (24 h). The modeling time is 28 days. The different stressors were randomly distributed, with an interval of seven days between repetitions. All stressors were administered four times within 28 days [16].

### 2.5. Chronic Constriction Injury of the Sciatic Nerve Model

After 28 days of chronic unpredictable mild stress, chronic constriction injury of the sciatic nerve model was made in the latter 3 groups of rats. Before the operation, the rats were anesthetized with isoflurane gas and placed on the operating table of the animals in the prone position. Remove the hair in the middle of the left thigh of rats, cut open the skin in the outer margin of the thigh femur, blunt and separate the muscles, and expose the sciatic nerve trunk. Wrap the 4-0 chrome gut around the middle of the sciatic nerve stem and tie a knot. Tighten the knot at a constant speed until it is just on either side of the nerve stem, creating a slight compression. Tie the second knot carefully to avoid any effect on the tightness of the first knot. In this way, the sciatic nerve trunk is evenly three knots with an interval of about 1 mm [17]. The skin wound was nailed with metal nails to prevent rats from gnawing. Each operator is fixed to ensure that the ligation force is comparable [18].

### 2.6. Intervention

After modeling, the taVNS and EA groups were treated with an electrical stimulator for 28 consecutive days. The intervention time was from 15:00 to 17:00 every day, and each time the electrical stimulation lasted 30 min. The intensity and frequency of electrical stimulation were set at 2 mA and 15 Hz. The waveform was selected as a disperse-dense wave. The intervention was operated under anesthesia with isoflurane gas. The taVNS group was connected with a positive and negative electrode self-adsorption conductive magnet which was noninvasively fixed in the bilateral cavity of the auricular concha of rats. The auricle of rats was observed to maintain slight vibration. If there was no vibration, a cotton swab was used to wipe the auricular concha with normal saline to enhance the conductive effect. The EA was inserted perpendicularly into the skin 5 mm apart at bilateral ST 36 in the EA group. ST 36 locates 5 mm below the humeral head [19]. The stimulation intensity, frequency, and waveform were the same as those of the taVNS group.

### 2.7. Sucrose Preference Test

All the rats were singly housed in a cage when the sucrose preference test began. Two bottles of 1% sucrose solution were placed in each cage at the same time for 24 h, and the animals were trained to adapt to drink sucrose water. Replace one of the bottles with pure water for the next 24 hours. After 23 h of food and water deprivation, each cage was given two bottles of water quantified in advance: one bottle of 1% sucrose water and one bottle of pure water. Sucrose and water bottles were placed in randomly assigned sides of the cage. After 60 min, remove two bottles and weigh them. The results of sucrose preference test were calculated according to the following equation: sucrose solution (g)/(sucrose solution (g) + water (g)) × 100%.

### 2.8. Mechanical Withdrawal Threshold

The mechanical pain threshold was measured by using the von Frey filaments. The rat was placed in the plastic cages on a perforated metal platform to habituate the environment for one hour the day before the first test and no measurements were made. Allow the rat to adapt to the environment for 15 min before each test. The stimulation force of von Frey filaments can provide a range of 0.008 g to 300 g. The Von Frey filaments were used to vertically stimulate the intermetatarsal bones of the 4th and 5th posterior feet of the rats. Brisk withdraw or paw flinching was considered as the effective stimulation which was recorded. The bilateral hind paw mechanical withdrawal threshold was tested three times, and the average values were calculated.

### 2.9. Thermal Stimulation Latency

The thermal stimulation latency was measured by using the Plantar Analgesia Meter. Each rat was placed in the individual plexiglass enclosure compartment on the glass surface to habituate the environment for one hour the day before the first test, and no measurements were made. Allow the rat to adapt to the environment for 5 min before each test. The thermal stimulus was emitted from a movable radiant heat source under the glass surface and was focused on the plantar surface of the hind paw. The source output temperature was set at 52°C. The cutoff time was set at 25 s to prevent potential tissue damage caused by continuous heating. Move the trigger with both hands, so that the radiant heat source is focused on the 4th and 5th tibia of the hind paw of the rat. Press the trigger button to heat the rat's foot. When the rat is withdrawing the paw, the instrument automatically records the latency time. Bilateral hind paw thermal withdrawal latencies were tested three times, and the average values were calculated.

### 2.10. ELISA Analysis

The plates were coated with the TNF-*α* and SP antigen (100 *μ*l/well) and incubated overnight at 4°C. The plates were washed with PBS-0.05% Tween20 (PBST) and blocked with PBST-1% BSA (200 *μ*l/well) at 37°C for one hour. The plates were washed, and the plasma samples diluted to different multiples were added and incubated at 37°C for two hours. The plates were washed, and the rabbit antirat IgG (H+L) was added and incubated at 37°C for one hour. The plates were washed, and a chromogenic solution was allowed to react for 20 min. The optical density (OD) value of each well was measured using a microplate reader at wavelengths of 562 nm. The standard curve was prepared according to the standard solution and corresponding OD value, and thus, the concentrations of TNF-*α* and SP of each sample could be calculated.

### 2.11. Western Blot Analysis

The total protein of brain samples was extracted using the extraction kit according to the manufacturer's instructions and analyzed with a bicinchoninic acid (BCA) protein concentration assay kit. Proteins (30 *μ*g/well) and protein ladder were separated by gel electrophoreses running on PowerPac HC Power Supply for approximately 20 min at 90 V in running buffer (250 mM Tris base, 2.5 M glycine, 1% SDS, pH 8.3) and transferred to polyvinylidene difluoride (PVDF) membranes at 300 mA for 90 min. The membranes were blocked with 5% nonfat dry milk in TBST (TBS containing 20% Tween-20, pH 7.5) for 1 h at room temperature. The primary antibodies (1 : 1000 dilution for TNF-*α*) were then incubated overnight at 4°C in TBST with 5% nonfat dry milk. After three 10-min washes in TBST, the secondary antibody was incubated in a 1 : 10000 dilution in TBST with 5% nonfat dry milk for 40 min at room temperature followed by three 10-min washes in TBST. The membranes were exposed to clarity enhanced chemiluminescence (ECL) reagent for 3 min at room temperature. The detection of immunoreactive bands was performed by image scan using a Gel Image system ver.4.00.

### 2.12. Statistical Analysis

The data were analyzed by using SPSS version 22.0 (SPSS Inc., Chicago, IL, USA) and GraphPad Prism 5.0 (GraphPad Software Inc., San Diego, CA, USA). The data is expressed as mean ± standard deviation. The paired *t*-test was used for the before-and-after comparison of data in the group. One-way analysis of variance was used for comparison of data between the groups. A value of *P* < 0.05 was considered statistically significant.

## 3. Results

### 3.1. The Weight in Each Group at Different Time Points

There was no significant difference in the weight between the four groups at -28 day (*P* > 0.05). Compared with the control group, the weight decreased significantly in the taVNS, EA, and model groups at -14 day and 0 day (*P* < 0.01), indicating the weight of rats were affected by CUMS. Compared with the model group, the weight decreased significantly in the taVNS group at 28 day (*P* < 0.05). The weight decreased in the taVNS group at 28 day compared with the EA group, but no statistical difference was found between them (*P* > 0.05) (Table 1).

### 3.2. Sucrose Preference in Each Group at Different Time Points

There was no significant difference in the sucrose preference between the four groups at -28 day (*P* > 0.05). Compared with the control group, the sucrose preference decreased significantly in the taVNS, EA, and model groups at 0 day (*P* < 0.05), suggesting that the CUMS model has been successfully built. Compared with the model group, the sucrose preference increased significantly in the taVNS group at 28 day (*P* < 0.05), the sucrose preference increased in the EA group at 28 day, but no statistical difference was found between them (*P* > 0.05). The difference in sucrose preference between the taVNS and model groups was similar to those between the EA and model groups (*P* > 0.05) (Table 2).

### 3.3. Mechanical Withdrawal Threshold in Each Group at Different Time Points

There was no significant difference in the mechanical withdrawal threshold between the four groups at -28 day (*P* > 0.05). Compared with the control group, the mechanical withdrawal threshold decreased significantly in the taVNS, EA, and model groups at 0 day (*P* < 0.001). Compared with the model group, the mechanical withdrawal threshold increased in the EA and taVNS groups at 28 day, but no statistical difference was found between them (*P* > 0.05). Compared with the EA group, the mechanical withdrawal threshold decreased in the taVNS group at 28 day, but no statistical difference was found between them (*P* > 0.05) (Table 3).

### 3.4. Thermal Stimulation Latency in Each Group at Different Time Points

There was no significant difference in the thermal stimulation latency between the four groups at -28 day (*P* > 0.05). Compared with the control group, the thermal stimulation latency decreased significantly in the taVNS, EA, and model groups at 0 day (*P* < 0.05). Compared with the model group, the thermal stimulation latency increased significantly in the EA and taVNS groups at 28 day (*P* < 0.05). Compared with the EA group, the thermal stimulation latency decreased in the taVNS group at 28 day, but no statistical difference was found between them (*P* >0.05) (Table 4).

### 3.5. The Concentration of TNF-*α* and SP in Plasma for Each Group

**Figure 2:**
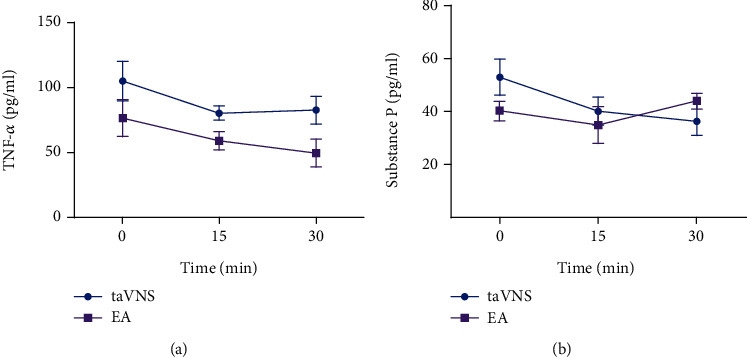
Comparison of plasma concentration of TNF-*α* and SP in taVNS and EA groups before, during, and after intervention. (a) Comparison of plasma concentration of TNF-*α* in taVNS and EA groups before, during, and after intervention. (b) Comparison of plasma concentration of SP in the taVNS and EA groups before, during, and after intervention.

Compared with the control group, the plasma concentration of TNF-*α* increased significantly in the model group at 28 day (*P* < 0.05). Compared with the model group, the TNF-*α* level decreased significantly in the EA group at 28 day (*P* < 0.01), the concentration of TNF-*α* decreased in the taVNS group at 28 day, but no statistical difference was found between them (*P* > 0.05) (Table 5). Compared with that at 0 min, the concentration of TNF-*α* in plasma decreased in the taVNS and EA groups at 15 and 30 min, but no statistical difference was found between them (*P* > 0.05). Compared with that at 15 min, the concentration of TNF-*α* decreased continuously in the EA group at 30 min, but no statistical difference was found between them (*P* > 0.05). There was a large difference between 0 min and 30 min in the EA group than those in the taVNS group (Figure 2(a)).

Compared with the control group, the concentration of SP in plasma decreased in the model group and taVNS group at 28 day, but no statistical difference was found between them (*P* > 0.05). The concentration of SP was higher in the EA group compared with the other three groups at 28 day, but no statistical difference was found between them (*P* > 0.05) (Table 5). Compared with that at 0 min, the concentration of SP decreased continuously in the taVNS group at 15 and 30 min, but no statistical difference was found between them (*P* > 0.05). Compared with that at 0 min, the plasma concentration of SP in the EA group decreased at 15 min and increased at 30min, but no statistical difference was found between them (*P* > 0.05) (Figure 2(b)).

### 3.6. The Expression of TNF-*α* in the PFC, Hippocampus, Amygdala, and Hypothalamus for Each Group at 28 Day

**Figure 3:**
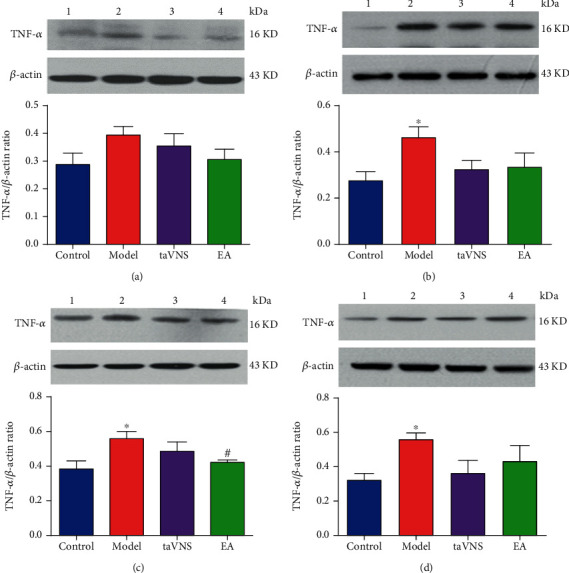
Expressions of TNF-*α* in PFC, hippocampus, amygdala, and hypothalamus. (a) Comparison of the TNF-*α* expression in the PFC for each group at 28 day. (b) Comparison of the TNF-*α* expression in the hippocampus for each group at 28 day. (c) Comparison of the TNF-*α* expression in the amygdala for each group at 28 day. (d) Comparison of TNF-*α* expression in the hypothalamus for each group at 28 day. 1: control group; 2: model group; 3: taVNS group; 4: EA group. ^∗^*P* < 0.05, vs. the control group. ^#^*P* < 0.05, vs. the model group.

Compared with the control group, the expression level of TNF-*α* in the hippocampus, amygdala, and hypothalamus increased significantly in the model group (*P* < 0.05). The expression level of TNF-*α* in PFC increased in the model group, but no statistical difference was found between them (*P* = 0.058). Compared with the model group, the expression level of TNF-*α* in the amygdala decreased significantly in the EA group (*P* < 0.05), the expression level of TNF-*α* in the hypothalamus and hippocampus decreased in the taVNS group, but no statistical difference was found between them (*P* = 0.054, 0.052). The expression level of TNF-*α* in the PFC, hypothalamus, and hippocampus decreased in the EA group, but no statistical difference was found between them (*P* > 0.05). Compared with the EA group, the expression level of TNF-*α* in the PFC and amygdala were higher in the taVNS group (*P* > 0.05), the expression level of TNF-*α* in the hippocampus and hypothalamus was lower in the taVNS group, but no statistical difference was found between them (*P* > 0.05) (Figure 3).

## 4. Discussion

Our researches have shown that taVNS is a promising potential treatment that can improve the severity of major depression [20–22] and taVNS can relieve neuropathic pain in Zucker Diabetes Fat rats by promoting melatonin secretion [18]. Acupuncture is recognized worldwide as a treatment with analgesic effect [23]. It is reported that EA at ST 36 can relieve the neuropathic pain induced by CCI [24]. Moreover, EA at DU 20 and ST 36 has a therapeutic effect on depression [25].

Based on previous research, we choose taVNS and EA at ST 36 as the intervention for comorbidity of depression and pain in our experiment. Compared to the general drug treatment, taVNS and EA have the advantages of obvious treatment effect, low treatment cost, convenient operation, and safety. In our experiment, we found that both taVNS and EA at ST 36 can improve the depressive behavior and relieve chronic pain in rats with depression-chronic pain comorbidity after 28 consecutive days of intervention. In comparison, taVNS is good at improving depression-like behavior, and EA at ST 36 is good at relieving the pain symptoms. No adverse reactions occurred in the taVNS and EA groups during the intervention, indicating the treatments in the two groups were safe.

In our experiment, we also found induction and alleviation of depression-like behavior and chronic somatic pain symptoms was closely associated with the change of TNF-*α* level in the peripheral blood and brain regions, suggesting the inflammatory and immune processes play an important role in the biological mechanisms of depression-chronic somatic pain comorbidity. In the inflammatory mechanism of comorbidity of depression and pain, the peripheral proinflammatory cytokines can access the brain and activate local CNS inflammatory networks to affect the function of neurotransmitters involved in the pathophysiology of depression and pain [9]. To be specific, the peripheral production of TNF-*α*, IL-1, and IL-6 by monocytes results in a subsequent production of TNF-*α* and other mediators in the brain via toll-like receptor 4 (TLR4) present on circumventricular organs and peripheral vagal nerve afferents, leading to the activation of microglia. Activated microglia are the main source of TNF-*α* within the brain, while neuronal cells and astrocytes can produce it at a lower level. Thus, the cross-talk between peripheral immune cells and immune cells in the CNS may induce a positive feedback loop that further increases the production of TNF-*α* and other proinflammatory cytokines [9, 26, 27]. In our experiment, we found that TNF-*α* was increased in the PFC, hippocampus, amygdala, hypothalamus, and plasma in rats with depression-chronic pain comorbidity. The change of TNF-*α* in the brain was in accordance with those in plasma, indicating the probable existence of crosstalk between the peripheral immune cells and immune cells in the CNS.

The cholinergic anti-inflammatory pathway (CAP) is a physiological mechanism whereby the CNS regulates or inhibits local or systemic inflammatory response with cholinergic nerves and their neurotransmitters [28]. It is an endogenous anti-inflammatory pathway that links the nervous system and the immune system via the vagus nerve. The CAP can be activated by VNS or cholinergic agonists [29]. Ach is a neurotransmitter mainly released by vagus nerve endings. And *α*7nAChR is a key protein of signals triggered by VNS to induce the endogenous CAP [28]. Ach activates *α*7nAChR on macrophages, lymphocytes, and other inflammatory cells, regulating the synthesis and release of inflammatory cytokines and relieving the systemic inflammatory reaction [30]. The anti-inflammatory effects of taVNS and EA at ST 36 are closely related to the vagus-mediated cholinergic pathway. Previous studies have shown that EA at ST 36 also can reduce the serum TNF-*α* level in septic rats. Moreover, abdominal vagotomy or *α*7nAChR inhibitor can reverse the suppressive role of EA [31]. It proves that the anti-inflammatory role of EA at ST 36 is likely to depend on an intact vagus nerve and might exert its effects by the cholinergic *α*7nAChR [32]. EA at ST 36 activates the somatic fiber endings around ST 36 points, which send the acupuncture signals to the spinal cord via somatic sensory nerve fibers. In the spinal cord, the nerve impulses are transmitted to the NTS. After relayed and integrated by NTS, the nerve impulses activate CAP via efferent vagus nerve [33, 34]. Auricular concha is the only region on the surface of mammals where vagal afferent fibers are distributed. Nerve impulses can be transmitted to the NTS relay along the auricular branches of the vagus nerve, and then exert cholinergic anti-inflammatory effects via the efferent vagus nerve [33, 35]. Our experimental results showed that EA at ST 36 and taVNS have their anti-inflammatory effect mainly by decreasing TNF-*α* in plasma and brain regions. Among them, EA at ST 36 plays a more significant role.

In addition to TNF-*α*, SP is also an indicator of our study. SP is an eleven-amino acid long neuropeptide, widely distributed in the CNS and peripheral nervous system [36, 37]. It is a member of the tachykinin family and is involved in many biological processes, including nociception and neurogenic inflammation [38, 39]. In the aspect of nociception, pain-sensing fibers (nociceptors) release SP to increase pain sensitivity through its actions in the dorsal horn of the spinal cord. SP transmits and integrates nociceptive signals; accumulating studies found that SP also has an antinociceptive effect [40, 41]. In the aspect of inflammation, SP plays a critical role in its ability to stimulate and/or modulate the production of various cytokines by a wide range of immune cells [39]. We found SP showed a tendency to decline before, during, and after taVNS. However, the results of the SP content in the four groups showed confusion at 28 day, which cannot be explained for a reasonable reason. The only reason may be that the plasma SP was stored in the refrigerator at -80°C for too long before the detection, resulting in the change of the content of SP during the storage process. In the future, we need to strictly control each step of SP detection to ensure the reliability of the experimental data. What is more, the lack of pathological images in the experiment is unable to provide the evidence of microglia activation in related brain regions. In the future, we need to use morphological methods to observe the activation of immune cells in peripheral plasma and related brain regions. Fortunately, we have found the important role of TNF-*α* in the inflammatory mechanism of depression-chronic somatic pain comorbidity, which lays the foundation for TNF-*α* regulator as a new drug target for the treatment of the disease. At the same time, we also found that EA at ST 36 and taVNS can improve the depression and relieve chronic somatic pain in model rats, which provides new and effective treatment methods for the disease. In a word, we need a large sample size, multi-indicators experiment to further research the anti-inflammatory mechanism of acupuncture in the comorbidity of depression and pain in the future.

## 5. Conclusion

CUMS combined CCI can induce depression-like behavior and chronic somatic pain disorders under solitary care for 28 consecutive days. After 28 consecutive days of intervention, both taVNS and EA at ST 36 can improve depression-like behavior and relieve chronic somatic pain. Compared with the control group, the levels of TNF-*α* in plasma, PFC, hippocampus, hypothalamus, and amygdala increased in rats with depression-chronic somatic pain comorbidity, and the elevated expression of TNF-*α* can be downregulated by taVNS and EA at ST 36.

## Figures and Tables

**Table 1:** Comparison of weight in each group at different time points ($\bar{x}\pm s$).

Group	-28 day		-14 day		0 day		14 day		28 day	
	*n*	Weight (g)	*n*	Weight (g)	n	Weight (g)	*n*	Weight (g)	*n*	Weight (g)
Control	10	219.4 ± 15.9	10	341.3 ± 30.3	8	386.0 ± 40.5	8	450.4 ± 42.7	7	459.7 ± 47.4
Model	10	219.0 ± 11.8	10	279.9 ± 18.0^∗∗∗^	8	338.6 ± 32.5^∗∗^	8	431.7 ± 43.4	8	452.1 ± 46.7
taVNS	10	213.0 ± 11.4	10	271.5 ± 23.3^∗∗∗^	8	319.6 ± 25.4^∗∗∗^	8	396.5 ± 25.3^∗^	8	406.5 ± 26.5^∗^^#^
EA	10	220.0 ± 13.2	10	285.5 ± 15.2^∗∗∗^	8	340.1 ± 28.2^∗∗^	8	409.5 ± 43.4^∗^	7	434.1 ± 29.8

At 0 day, two rats were removed from each group. At 24 day, two rats from the control and EA groups died when experimenters took blood from the caudal vein. ^∗∗∗^*P* < 0.001, vs. the control group; ^∗∗^*P* < 0.01, vs. the control group; ^∗^*P* < 0.05, vs. the control group; ^#^*P* < 0.05, vs. the model group.

**Table 2:** Comparison of sucrose preference in each group at different time points ($\bar{x}\pm s$).

Group	-28 day		0 day		28 day	
	*n*	SP (%)	*n*	SP (%)	*n*	SP (%)
Control	10	0.62 ± 0.19	8	0.76 ± 0.12	7	0.80 ± 0.12
Model	10	0.57 ± 0.22	8	0.59 ± 0.12^∗^	8	0.63 ± 0.13
taVNS	10	0.71 ± 0.21	8	0.60 ± 0.15^∗^	8	0.81 ± 0.16^#^
EA	10	0.62 ± 0.21	8	0.60 ± 0.16^∗^	7	0.80 ± 0.25

SP: sucrose preference. ^∗^*P* < 0.05, vs. the control group; ^#^*P* < 0.05, vs. the model group.

**Table 3:** Comparison of the mechanical withdrawal threshold in each group at different time points ($\bar{x}\pm s$).

Group	-28 day		-14 day		0 day		14 day		28 day	
	*n*	MWT (g)	*n*	MWT (g)	*n*	MWT (g)	*n*	MWT (g)	*n*	MWT (g)
Control	10	8.0 ± 1.6	10	6.7 ± 3.9	8	6.8 ± 1.8	8	6.3 ± 2.3	7	7.4 ± 2.2
Model	10	7.7 ± 2.7	10	4.9 ± 2.3	8	2.3 ± 2.8^∗∗∗^	8	3.6 ± 2.9^∗^	8	4.6 ± 2.9^∗^
taVNS	10	7.8 ± 2.6	10	5.2 ± 2.5	8	1.2 ± 1.3^∗∗∗^	8	3.4 ± 2.1^∗^	8	5.3 ± 2.6
EA	10	8.4 ± 1.8	10	5.8 ± 2.4	8	1.2 ± 0.6^∗∗∗^	8	4.0 ± 2.6	7	6.0 ± 2.3

MWT: mechanical withdrawal threshold. ^∗∗∗^*P* < 0.001, vs. the control group; ^∗^*P* < 0.05, vs. the control group.

**Table 4:** Comparison of thermal stimulation latency in each group at different time points ($\bar{x}\pm s$).

Group	-28 day		14 day		0 day		14 day		28 day	
	*n*	TSL (sec)	*n*	TSL (sec)	*n*	TSL (sec)	*n*	TSL (sec)	*n*	TSL (sec)
Control	10	12.03 ± 3.42	10	12.42 ± 1.41	8	11.82 ± 2.76	8	11.14 ± 2.25	7	12.12 ± 2.87
Model	10	11.30 ± 2.85	10	11.52 ± 2.46	8	7.57 ± 2.08^∗∗^	8	9.56 ± 1.60	8	9.47 ± 1.27^∗^
taVNS	10	11.17 ± 2.72	10	11.26 ± 2.57	8	8.84 ± 2.26^∗^	8	10.58 ± 2.20	8	11.88 ± 1.30^#^
EA	10	10.73 ± 2.98	10	9.94 ± 1.92^∗^	8	8.76 ± 2.03^∗^	8	10.07 ± 3.08	7	12.20 ± 1.60^##^

TSL: thermal stimulation latency. ^∗∗^*P* < 0.01, vs. the control group; ^∗^*P* < 0.05, vs. the control group; ^##^*P* < 0.01, vs. the model group; ^#^*P* < 0.05, vs. the model group.

**Table 5:** Comparison of plasma concentration of TNF-*α* and SP in each group at 28 day ($\bar{x}\pm s$).

Group	*n*	TNF-*α* (pg/ml)	SP (pg/ml)
Control	7	29.94 ± 32.11	114.86 ± 58.77
Model	8	76.24 ± 39.74^∗^	85.34 ± 44.63
taVNS	8	49.88 ± 29.70	84.34 ± 54.84
EA	7	25.16 ± 26.99^##^	124.08 ± 45.24

^∗^ *P* < 0.05, vs. the control group; ^##^*P* < 0.01, vs. the model group.

## Data Availability

The data used to support the findings of this study are available from the corresponding author upon request.
